# Non-linear relationship between albumin-corrected calcium and 30-day in-hospital mortality in ICU patients: A multicenter retrospective cohort study

**DOI:** 10.3389/fendo.2022.1059201

**Published:** 2022-12-21

**Authors:** Xun Qin, Ji Cen, Haofei Hu, Xinglin Chen, Zhe Wei, Qijun Wan, Rong Cao

**Affiliations:** ^1^ Department of Nephrology, Hechi People’s Hospital, Hechi, China; ^2^ Department of Nephrology, Shenzhen Second People’s Hospital, Shenzhen, Guangdong, China; ^3^ Department of Nephrology, The First Affiliated Hospital of Shenzhen University, Shenzhen, Guangdong, China; ^4^ Department of Geriatrics, Union Hospital, Tongji Medical College, Huazhong University of Science and Technology, Wuhan, China; ^5^ Department of Epidemiology and Biostatistics, Empower U, X&Y Solutions Inc., Boston, MA, United States

**Keywords:** albumin-corrected calcium, hypocalcemia, hypercalcemia, hospital mortality, ICU patients, non-linear relationship, multicenter study

## Abstract

**Objective:**

Albumin-corrected calcium is usually calculated to reflect the real serum calcium level of the whole body by physicians. However, studies on the association between albumin-corrected calcium and 30-day in-hospital mortality in Intensive Care Unit (ICU) patients are rare. The purpose of our study was to explore the association between baseline albumin-corrected calcium and 30-day in-hospital mortality in the American ICU population.

**Methods:**

A multicenter retrospective cohort study of 102,245 ICU patients in the eICU-CRD v2.0 from the USA during 2014–2015 was performed. The average age was 63.7 ± 16.9 years, of which 55,313 (53.7%) were men and 47,758 (46.3%) were women. The association between albumin-corrected calcium and 30-day in-hospital mortality was analyzed by Cox proportional-hazards regression, smooth curve fitting, piecewise linear regression, subgroup analyses, and a series of sensitivity analyses.

**Results:**

We found that among ICU patients with calcium abnormalities, more than 95% were mild hypocalcemia or mild hypercalcemia. The risk of 30-day in-hospital mortality will increase by 10% in the ≥7.5–< 8.5 mg/dl subgroup (OR=1.1, 95% CI 1.0–1.3) or 20% in the ≥10.3–<12 mg/dl subgroup (OR=1.2, 95% CI 1.1–1.3) when the albumin-corrected calcium level increases by 1 mg/dl. Additionally, the relationship between albumin-corrected calcium and 30-day in-hospital mortality was U shaped; the inflection point was 8.9 mg/dl (log likelihood ratio test P = 0.005). Finally, after a series of sensitivity analyses, we found that the relationship between albumin-corrected calcium and 30-day in-hospital mortality remained significant.

**Conclusion:**

In a large nationally representative cohort of ICU patients, abnormalities in albumin-corrected calcium, particularly slight hypocalcemia or slight hypercalcemia, were associated with an increased 30-day in-hospital mortality risk, and yet the findings in this study need to be further confirmed by prospective studies.

## Introduction

Electrolyte metabolism disorders, including abnormal serum sodium and potassium concentrations, are highly prevalent among ICU patients, and they are included in the APACHE II prognostic system to evaluate the severity and clinical outcome of these patients (1). Abnormal serum calcium, although not included in the APACHE II prognostic system, is very common among ICU patients (2, 3). The disorders of calcium metabolism can affect the excitability of the neuromuscular system, leading to cardiac arrhythmia, multiorgan dysfunction, and even comatose and cardiac arrest (4). However, clinicians generally do not pay enough attention to serum calcium abnormalities, and only severe hypocalcemia or hypercalcemia is actively treated and intervened as we all know that an extremely high or low serum calcium level has been predicted to increase the risk of mortality, while mild hypocalcemia and hypercalcemia are not so actively intervened because the evidence for the effect of mild calcium abnormalities on mortality is relatively scarce. Thus, what is the incidence and distribution of serum calcium abnormalities in the ICU? In addition, what is the impact of mild hypercalcemia and hypocalcemia on the mortality of ICU patients?

When we mention serum calcium, we usually refer to total calcium, ionized calcium, and albumin-corrected calcium (5).Total calcium is affected by the pH and serum albumin level; thus, it is not very accurate (6); Additionally, ionized calcium, which can accurately reflect the functional calcium level, is not routinely tested (4). Therefore, clinicians often use albumin-corrected calcium to evaluate the ionized calcium level (7). As previously reported, abnormal calcium concentration could increase the mortality of patients. Hypocalcemia was an independent risk factor for long-term mortality in patients with acute pulmonary embolism (8). Serum calcium was an independent risk factor for predicting the in-hospital mortality of acute myocardial infarction patients (9). However, these studies mostly focused on total calcium or ionized calcium and mainly focused on hypocalcemia. Hypercalcemia was very common, but relevant studies were relatively lacking, and multicenter large sample studies on albumin-corrected calcium and mortality in ICU patients are limited.

We hypothesized that even high- and low albumin–corrected calcium levels are associated with a higher risk of 30-day in-hospital mortality in ICU patients. Therefore, our study intends to analyze the eICU-CRD v2.0 database in order to 1) identify the prevalence and distribution of the calcium abnormalities of ICU patients in in the United States and 2) investigate the effect of albumin-corrected calcium levels, particularly mild hypocalcemia and hypercalcemia, on the 30-day in-hospital mortality of ICU patients by using a multicenter observational cohort study.

## Methods

### Data source

This study is a multicenter retrospective cohort study. We obtained the data for this study from the eICU Collaborative Research Database v2.0 (eICU-CRD v2.0). The database includes 200,859 ICU patients’ medical records across 335 ICUs at 208 hospitals (academic and non-academic) in the USA during 2014–2015. The original database is a collection of 31 tables, including demographics, clinical history, vital signs, laboratory data, diagnoses, and treatments (10).

### Population selection criteria

**Figure 1:**
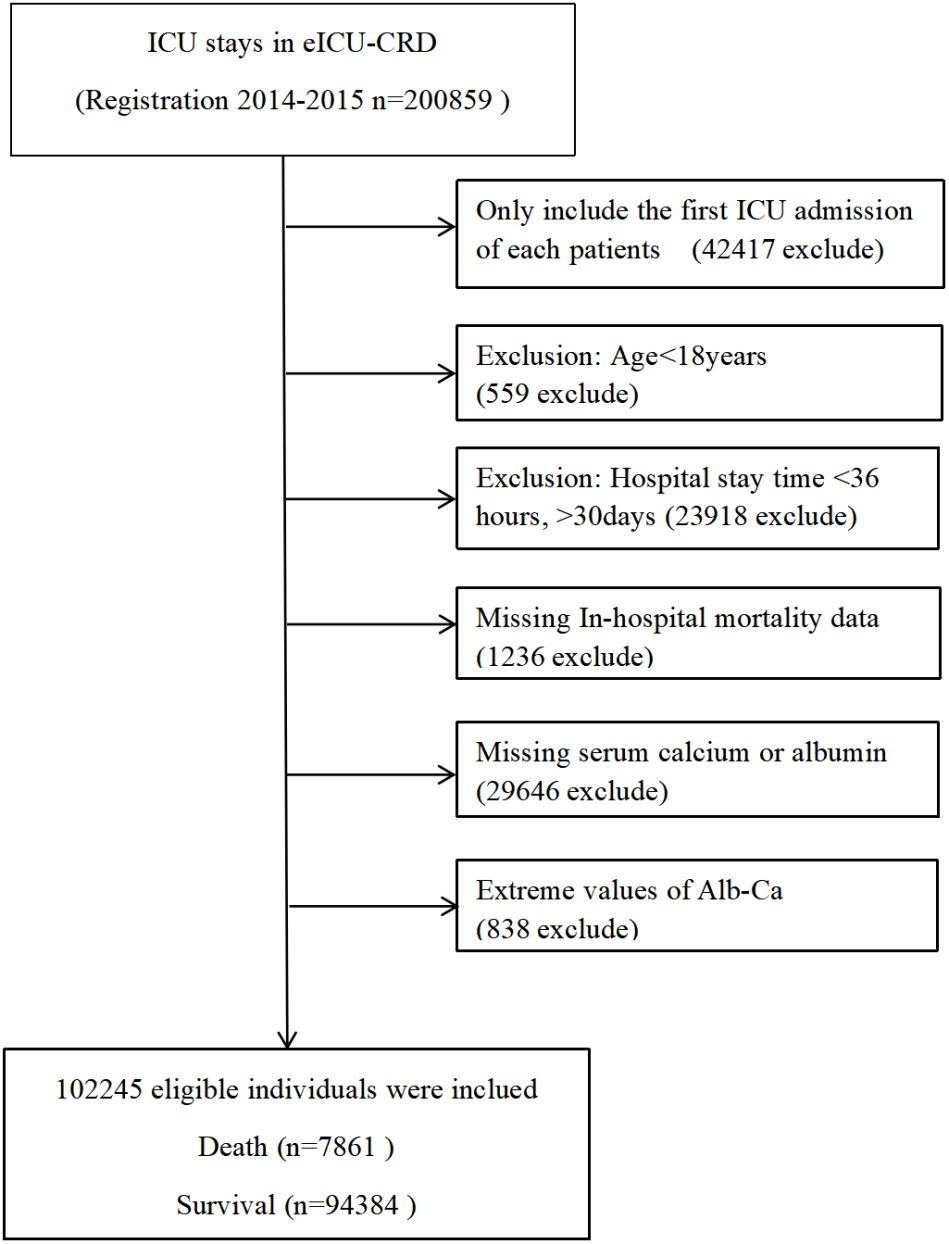
The non-linear relationship between albumin-corrected calcium and 30-day in-hospital mortality.The relationship between albumin-corrected calcium and 30-day in-hospital mortality. A non-linear relationship between them was detected after adjusting for age, gender, body mass index, ethnicity, cardiac arrest, gastrointestinal bleeding, diabetes mellitus, cancer, lactic acid, alanine aminotransferase, and serum albumin.

A total of 102,245 eligible individuals were enrolled in this study according to the following exclusion criteria: (1) Non-first-time ICU admission: n = 42,417; (2) <18 years old: n = 559; (3) ICU stay <48 h and >30 days: n = 23,918; (4) missing in-hospital mortality data: n = 1,236; and (5) missing serum calcium or albumin data after ICU admission: n = 29,646. In addition, the albumin-corrected calcium was calculated using the following equation: albumin-corrected calcium (mg/dl) = serum total calcium (mg/dl) + 0.8×[4.0-serum albumin (g/dl)]. Individuals were excluded if the albumin-corrected calcium value was an extreme value (the extreme value = mean ± 3SD) (n = 838) (11). Finally, 102,245 participants (54,878 men and 47,367 women) were included in this analysis. **Figure 1** is the flow chart that described the study design and participant flow.

### Data extraction

The variables extracted from eICU-CRD v2.0 were as follows: 1) demographic characteristics: age, gender, ethnicity, and body mass index (BMI); 2) comorbidities: diabetes mellitus, ketoacidosis, hypertension, acute respiratory failure, chronic obstructive pulmonary disease (COPD), acute myocardial infarction, atrial fibrillation, cardiac arrest, congestive heart failure, gastrointestinal bleeding, chronic kidney disease (CKD), end-stage renal disease (ESRD), sepsis, stroke, and cancer; 3) laboratory parameters: hemoglobin, platelet count, serum albumin, serum creatinine, serum magnesium, cholesterol, triglycerides, ALT, PH, and lactate; 4) scoring systems: Acute Physiology and Chronic Health Evaluation–IV (APACHE-IV) score; and 5) treatment: mechanical ventilation, nitroglycerin, glucocorticoids, vancomycin, carbapenem, and levofloxacin. The primary endpoint was 30-day in-hospital mortality. Serum calcium and serum albumin were tested on ICU admission, and the albumin-corrected calcium was calculated based on the baseline serum calcium and albumin according to the equation mentioned before. The baseline parameters were collected within the first 24 h after participant ICU admission.

### Statistical analysis

Albumin-corrected calcium was stratified into six groups: 1) <7.5 mg/dl; 2) 7.5–<8.5 mg/dl; 3) 8.5–<9.5 mg/dl; 4) 9.5–<10.3 mg/dl; 5)10.3–<12 mg/dl; and 6) ≥12 mg/dl. Continuous variables were presented as the means ± standard deviations (normal distribution) or medians (quartiles) (skewed distribution), and compared using one-way ANOVA or Kruskal–Wallis. Categorical variables were expressed as percentage and compared by the chi-square test.

The association between albumin-corrected calcium and 30-day in-hospital mortality was determined using the Cox proportional hazards model. Baseline albumin-corrected calcium was fitted as both continuous and categorical variables (stratified into six subgroups: <7.5 mg/dl; 7.5–<8.5 mg/dl; 8.5–<9.5 mg/dl; 9.5–<10.3 mg/dl; 10.3–<12 mg/dl; and ≥12 mg/dl). The third category (8.5–< 9.5 mg/dl) was considered as the control group to calculate the hazard ratios (HRs) and pertinent 95% confidence interval (CI) for the other albumin-corrected calcium subgroups. The results of unadjusted (crude model), minimally adjusted (model I) and fully adjusted (model II) are shown in **Table 1**. In model I, covariates were adjusted for age, gender, BMI, and ethnicity. In model II, we adjusted age, gender, BMI, ethnicity, cardiac arrest, gastrointestinal bleeding, diabetes mellitus, cancer, lactic acid, ALT, and serum albumin.

**Table 1:** Threshold effect analysis of albumin-corrected calcium and 30-day in-hospital mortality using piecewise linear regression.

Inflection point of Alb-Ca (mg/dl)	OR	95%CI	P-value
<8.9	0.9	0.8–1.0	0.094
≥8.9	1.1	1.1–1.2	<0.001

Effect: in-hospital mortality cause: albumin-corrected calcium.

Adjusted: age, gender, BMI, ethnicity; cardiac arrest; gastrointestinal bleeding; diabetes mellitus; cancer; lactic acid; ALT; serum albumin.

Alb-Ca, albumin-corrected calcium; OR, odds ratio; CI, confidence interval; ALT, glutamic pyruvic transaminase.

We conducted a series of sensitivity analyses to ensure the stability of the relationship between albumin-corrected calcium and 30-day in-hospital mortality. We replicated the multivariate Cox proportional hazards model in the population data sets in which cancer or ESRD patients were excluded. As albumin-corrected calcium was a continuous variable, we used the smooth curve fitting model and generalized additive model (GAM) to identify the non-linear relationships between albumin-corrected calcium and 30 day in-hospital mortality. The two-piece wise linear regression model was performed to calculate the threshold effect of albumin-corrected calcium on 30-day in-hospital mortality in terms of the smoothing plot.

To evaluate the stability of the association between albumin-corrected calcium and 30-day in-hospital mortality, we performed subgroup analyses using stratified linear regression models, and the modifications and interactions of these subgroups were inspected by likelihood ratio tests. The subgroups were defined by age (<60 and ≥60 years), gender, BMI (<18.5 and ≥18.5, <23.9 and ≥23.9), ethnicity, cardiac arrest, gastrointestinal bleeding, diabetes mellitus, cancer, serum albumin (<3.5 g/dl and ≥3.5 g/dl), ALT (<40 and ≥40 U/L), and lactic acid (<1.7 and ≥1.7 mmol/L).

All of the analyses were performed with the statistical software package R (http://www.R-project.org, The R Foundation) and Empower Stats (http://www.empowerstats.com, X&Y Solutions, Inc., Boston, MA, USA). P-values less than 0.05 (two sided) were considered statistically significant.

## Result

### Demographics and characteristics of the participants

**Figure 2:**
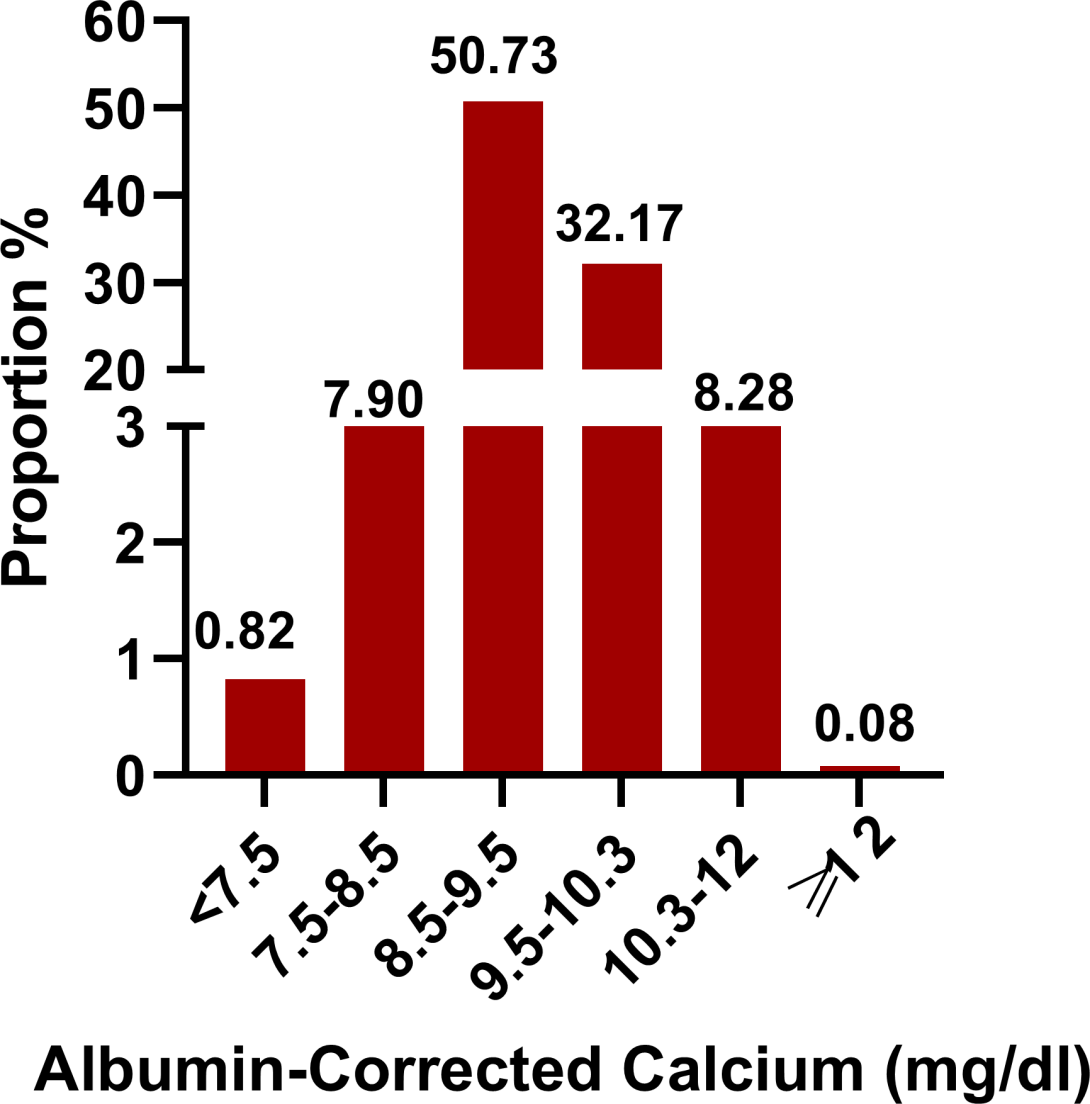
Distribution of albumin-corrected calcium. It shows a normal distribution according to stratified range of values.

Our study included 102,245 participants, of which 7,861 participants incurred death as shown in **Figure 1**. The albumin-corrected calcium value shows a normal distribution, ranging from 6.58 to 12.12, and, according to a stratified range of values, the abnormalities of albumin-corrected calcium occurred in 17.1% of the ICU population, of which mild calcium abnormalities (including mildly low calcium and mildly high calcium) accounted for more than 95% of the population within the calcium abnormalities, while severe calcium abnormalities accounted for less than 5%. The above data suggest that we should pay more attention to mild calcium abnormalities in clinical practice (**Figure 2**). In age stratification by 10 intervals, the albumin-corrected calcium values slightly increased with age, both in male and female patients. There was no significant difference in albumin-corrected calcium levels between men and women no matter what age subgroup they were in (**Supplementary Figure 1**).

The patient’s characteristics were layered through the tertiles of albumin-corrected calcium. **Table 2** compares the patient’s demographics, comorbidities, laboratory parameters, scoring systems, and treatment. The average age of the participants was 63.7 ± 16.9 years old; 53.7% of them were men, and the Caucasians had the highest percentage (76.5%). The highest albumin-corrected calcium group (≥12 mg/dl) had the highest mortality, APACHE-IV score, serum magnesium, and lactate values, and it had the most diabetes mellitus, acute respiratory failure, atrial fibrillation, cardiac arrest, chronic kidney disease, ESRD, sepsis, cancer, vancomycin, and carbapenem treatment population; furthermore, this group had the lowest BMI and serum albumin levels. However, the lowest albumin-corrected calcium group (<7.5 mg/dl) had the most gastrointestinal bleeding, ketoacidosis, and mechanical ventilation treatment population, and this subgroup had the lowest age, hemoglobin, serum magnesium, cholesterol, and platelet levels.

**Table 2:** The baseline characteristics of participants (N = 102,245).

Characteristic	Albumin-corrected calcium (mg/dl)						
	<7.5	≥7.5, <8.5	≥8.5, <9.5	≥9.5, <10.3	≥10.3, <12	≥12	P-value
Participants	841	8078	51876	32898	8472	80	
Age, years	56.6 ± 17.1	57.2 ± 18.0	62.9 ± 17.1	65.9 ± 16.0	66.7 ± 15.5	65.3 ± 13.2	<0.001
Gender—n (%)							<0.001
Male	463 (55.1%)	4,673 (57.9%)	29,367 (56.6%)	16,438 (50.0%)	3,898 (46.0%)	39 (48.8%)	
Female	377 (44.9%)	3,404 (42.1%)	22,502 (43.4%)	16,458 (50.0%)	4,573 (54.0%)	41 (51.2%)	
BMI (kg/m2)	27.6 ± 7.1	27.4 ± 7.2	27.9 ± 7.7	27.7 ± 7.9	27.1 ± 8.1	26.6 ± 8.8	<0.001
Ethnicity (%)							<0.001
Black	85 (10.3%)	733 (9.2%)	5,263 (10.2%)	4,280 (13.1%)	1,323 (15.7%)	18 (22.5%)	
Asian	22 (2.7%)	168 (2.1%)	998 (1.9%)	553 (1.7%)	116 (1.4%)	1 (1.2%)	
Caucasian	617 (74.8%)	6,086 (76.3%)	39,812 (77.5%)	24,740 (75.8%)	6,277 (74.6%)	55 (68.8%)	
Hispanic	33 (4.0%)	357 (4.5%)	2,151 (4.2%)	1,359 (4.2%)	309 (3.7%)	4 (5.0%)	
White	15 (1.8%)	133 (1.7%)	424 (0.8%)	191 (0.6%)	52 (0.6%)	1 (1.2%)	
Other	53 (6.4%)	499 (6.3%)	2743 (5.3%)	1,525 (4.7%)	340 (4.0%)	1 (1.2%)	
APACHE-IV score	48.2 ± 13.2	45.2 ± 14.2	45.3 ± 14.0	48.0 ± 13.5	50.8 ± 13.0	52.6 ± 12.8	<0.001
**Comorbid conditions (%)**							
Diabetes mellitus	82 (9.8%)	669 (8.3%)	5,327 (10.3%)	4,434 (13.5%)	1,262 (14.9%)	12 (15.0%)	<0.001
Ketoacidosis	46 (5.5%)	389 (4.8%)	1,539 (3.0%)	1,082 (3.3%)	380 (4.5%)	3 (3.8%)	<0.001
Hypertension	88 (10.5%)	877 (10.9%)	6,961 (13.4%)	4,781 (14.5%)	1,136 (13.4%)	11 (13.8%)	<0.001
ARF	198 (23.5%)	1,437 (17.8%)	7,695 (14.8%)	5,252 (16.0%)	1,655 (19.5%)	26 (32.5%)	<0.001
COPD	37 (4.4%)	394 (4.9%)	3,849 (7.4%)	3,066 (9.3%)	795 (9.4%)	3 (3.8%)	<0.001
AMI	32 (3.8%)	367 (4.5%)	3,202 (6.2%)	1,773 (5.4%)	349 (4.1%)	2 (2.5%)	<0.001
AF	48 (5.7%)	489 (6.1%)	4,299 (8.3%)	3,412 (10.4%)	953 (11.2%)	10 (12.5%)	<0.001
Cardiac arrest	57 (6.8%)	406 (5.0%)	1,420 (2.7%)	779 (2.4%)	274 (3.2%)	8 (10.0%)	<0.001
CHF	42 (5.0%)	463 (5.7%)	4,640 (8.9%)	3,506 (10.7%)	838 (9.9%)	7 (8.8%)	<0.001
GB	25 (3.0%)	195 (2.4%)	1,097 (2.1%)	542 (1.6%)	130 (1.5%)	2 (2.5%)	<0.001
CKD	19 (2.3%)	80 (1.0%)	629 (1.2%)	514 (1.6%)	159 (1.9%)	5 (6.2%)	<0.001
ESRD	54 (6.4%)	287 (3.6%)	1,295 (2.5%)	1,136 (3.5%)	461 (5.4%)	6 (7.5%)	<0.001
Sepsis	155 (18.4%)	1,133 (14.0%)	6,890 (13.3%)	5,816 (17.7%)	2,088 (24.6%)	33 (41.2%)	<0.001
Stroke	28 (3.3%)	295 (3.7%)	3,186 (6.1%)	1,859 (5.7%)	392 (4.6%)	1 (1.2%)	<0.001
Cancer	16 (1.9%)	145 (1.8%)	740 (1.4%)	492 (1.5%)	143 (1.7%)	5 (6.2%)	<0.001
**Laboratory data**							
Hb (g/dl)	11.4 ± 2.8	12.0 ± 2.7	12.2 ± 2.6	12.0 ± 2.5	11.8 ± 2.5	11.8 ± 2.8	<0.001
Platelet (10^9/L)	199.5 ± 89.8	207.0 ± 88.3	219.9 ± 88.0	236.8 ± 96.8	252.9 ± 107.4	259.3 ± 128.7	<0.001
ALB (g/dl)	3.2 ± 0.8	3.4 ± 0.7	3.4 ± 0.7	3.1 ± 0.7	2.8 ± 0.7	2.7 ± 0.7	<0.001
Creatinine (mg/dl)	1.3 (0.8–3.8)	1.0 (0.8–1.7)	1.0 (0.8–1.5)	1.1 (0.8–1.7)	1.3 (0.9–2.2)	1.6 (1.0–2.8)	<0.001
Mg (mg/dl)	1.7 ± 0.5	1.8 ± 0.4	1.9 ± 0.4	1.9 ± 0.4	1.9 ± 0.4	2.0 ± 0.5	<0.001
TC (mg/dl)	128.5 (93.5-169.2)	137.0 (110.0-173.0)	148.0 (118.0-181.0)	145.0 (115.0-179.0)	137.0 (108.0-172.0)	133.0 (99.0-199.0)	<0.001
TG (mg/dl)	132.0 (91.0-203.0)	118.0 (80.0-180.0)	110.0 (77.0-164.0)	110.0 (78.0-163.0)	117.0 (81.2-174.0)	124.0 (98.5-214.0)	<0.001
ALT (U/L)	30.0 (18.0-62.8)	29.0 (18.0-53.0)	25.0 (17.0-40.0)	24.0 (16.0-38.0)	23.0 (15.0-39.0)	22.5 (16.0-38.8)	<0.001
PH	7.3 ± 0.1	7.3 ± 0.1	7.4 ± 0.1	7.4 ± 0.1	7.4 ± 0.1	7.3 ± 0.1	<0.001
Lactate (mmol/L)	2.0 (1.2-3.7)	1.9 (1.2-3.3)	1.7 (1.1-2.8)	1.7 (1.1-2.8)	1.9 (1.2-3.0)	2.3 (1.4-3.2)	<0.001
**Treatment**							
Mechanical ventilation	341 (40.5%)	2,643 (32.7%)	12,118 (23.4%)	7,246 (22.0%)	2,146 (25.3%)	28 (35.0%)	<0.001
Nitroglycerin	10 (1.2%)	156 (1.9%)	1,646 (3.2%)	915 (2.8%)	152 (1.8%)	1 (1.2%)	<0.001
Glucocorticoids	34 (4.0%)	387 (4.8%)	3,125 (6.0%)	2,232 (6.8%)	542 (6.4%)	5 (6.2%)	<0.001
Vancomycin	65 (7.7%)	436 (5.4%)	2,607 (5.0%)	1,980 (6.0%)	686 (8.1%)	7 (8.8%)	<0.001
Carbapenem	16 (1.9%)	85 (1.1%)	505 (1.0%)	338 (1.0%)	117 (1.4%)	3 (3.8%)	<0.001
Levofloxacin	23 (2.7%)	212 (2.6%)	1,319 (2.5%)	1,001 (3.0%)	287 (3.4%)	1 (1.2%)	<0.001
Mortality	74 (8.8%)	651 (8.1%)	3,253 (6.3%)	2,745 (8.3%)	1,113 (13.1%)	25 (31.2%)	<0.001

Continuous data are expressed as mean ± SD or median (Q1–Q3).

Categorical data are expressed as n (%).

One-way ANOVA, Kruskall–Wallis test, or chi-square test.

BMI, body mass index; APACHE-IV score, Acute Physiology and Chronic Health Evaluation–IV score; ARF, acute respiratory failure; COPD, chronic obstructive pulmonary disease; AMI, acute myocardial infarction; AF, atrial fibrillation; CHF, congestive heart failure; GB, gastrointestinal bleeding; CKD, chronic kidney disease; ESRD, end-stage renal disease; Hb, hemoglobin; ALB, serum albumin; Mg, serum magnesium; TC, cholesterol; TG, triglyceride; ALT, alanine aminotransferase; Mortality, 30-day in-hospital mortality.

### Relationship between albumin-corrected calcium level and 30-day in-hospital mortality

In order to find which variables are possibly associated with 30-day in-hospital mortality, univariate analysis was performed (**Supplementary Table 1**). We found that 30-day in-hospital mortality was positively associated with the APACHE-IV score, diabetes mellitus, acute respiratory failure, COPD, acute myocardial infarction, atrial fibrillation, cardiac arrest, congestive heart failure, CKD, ESRD, sepsis, stroke, cancer, serum creatinine, serum magnesium, lactate, mechanical ventilation, glucocorticoids, vancomycin, carbapenem, and levofloxacin, whereas 30-day in-hospital mortality was negatively associated with ketoacidosis, hypertension, hemoglobin, serum albumin, pH, and nitroglycerin.

**Figure 3:**
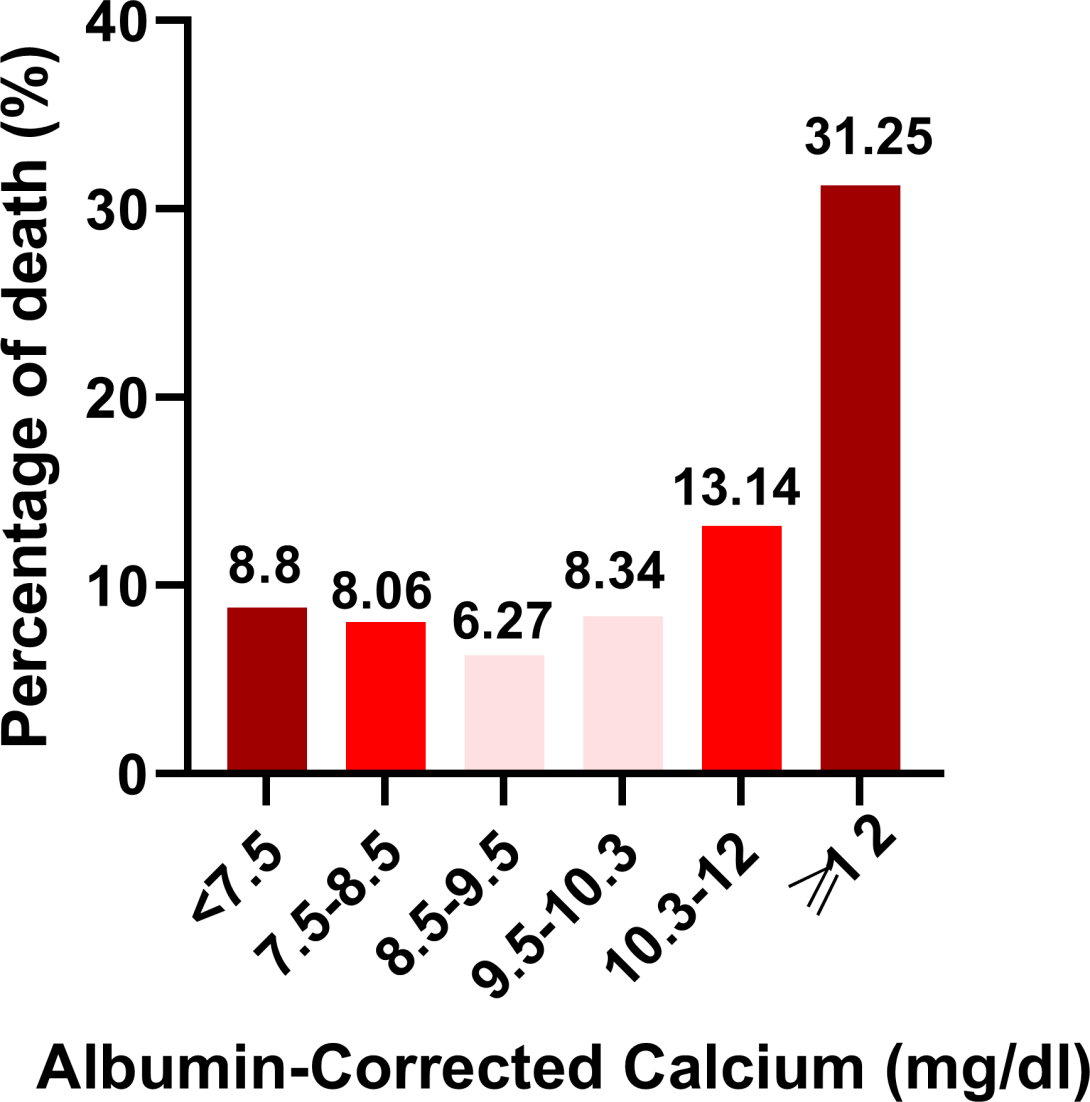
Histogram depicting percentage of death stratifed according to Albumin-Corrected Calcium (mgdl) categories.

As shown in **Figure 3**, the percentage of death was highest in the ≥12 mg/dl subgroup (31.25%), the second-highest group’s percentage of death was 13.14% in the 10.3–12 mg/dl subgroup. The percentage of death was lowest in the 8.5–9.5 mg/dl subgroup (6.27%). Univariate linear regression models were used to evaluate the associations between the albumin-corrected calcium level and 30-day in-hospital mortality. Meanwhile, we show the non-adjusted and adjusted models in **Table 3**. In the crude model, we found that the risk of 30-day in-hospital mortality will increase by 40% when the albumin-corrected calcium level increases by 1 mg/dl (OR = 1.4, 95% CI 1.3–1.4, P < 0.001). This association between the albumin-corrected calcium level and 30-day in-hospital mortality persisted despite adjustment for age, gender, BMI, and ethnicity (OR = 1.3, 95% CI 1.3–1.4, P < 0.001) (model I) or the inclusion of the above-mentioned baseline characteristics and cardiac arrest, gastrointestinal bleeding, diabetes mellitus, cancer, lactic acid, ALT, and serum albumin (model II) (OR = 1.1, 95%CI 1.0–1.1, P = 0.004).

**Table 3:** Relationship between albumin-corrected calcium (mg/dl) and 30-day in-hospital mortality in different models.

Variable	Crude model(OR, 95%CI, P)	Model I(OR, 95%CI, P)	Model II(OR, 95%CI, P)	GAM(OR, 95%CI, P)
Alb-Ca (mg/d)	1.4 (1.3, 1.4) <0.001	1.3 (1.2, 1.3) <0.001	1.1 (1.0, 1.1) 0.004	1.1 (1.0, 1.1) 0.004
Alb-Ca (tertiles) (mg/d)				
<7.5	1.4 (1.1, 1.8) 0.003	1.7 (1.3, 2.2) <0.001	1.0 (0.7, 1.4) 0.864	1.0 (0.7, 1.3) 0.831
≥7.5, < 8.5	1.3 (1.2, 1.4) <0.001	1.4 (1.3, 1.6) <0.001	1.1 (1.0, 1.3) 0.039	1.1 (1.0, 1.3) 0.060
≥8.5, < 9.5	Ref.	Ref.	Ref.	Ref.
≥9.5, < 10.3	1.4 (1.3, 1.4) <0.001	1.3 (1.2, 1.4) <0.001	1.1 (1.0, 1.1) 0.150	1.1 (1.0, 1.1) 0.141
≥10.3,< 12	2.3 (2.1, 2.4) <0.001	2.1 (2.0, 2.3) <0.001	1.2 (1.1, 1.3) <0.001	1.2 (1.1, 1.3) <0.001
≥12	6.8 (4.2, 10.9) <0.001	7.0 (4.3, 11.4) <0.001	2.1 (1.0, 4.3) 0.052	2.0 (1.0, 4.1) 0.063
P for trend	<0.001	<0.001	0.003	0.004

Crude model: we did not adjust other covariants.

Model I: we adjusted for age; gender; BMI; Ethnicity.

Model II: we adjusted age, gender, BMI, ethnicity; cardiac arrest; gastrointestinal bleeding; diabetes mellitus; cancer; lactic acid; ALT; serum albumin.

GAM: All variables listed in Model II were adjusted. However, continuous variables (age, BMI, lactic acid, ALT and serum albumin) were adjusted as non-linearity.

CI, confidence interval; OR, odds ratio; Alb-Ca, albumin-corrected calcium; ALT, glutamic pyruvic transaminase.

### Sensitivity analysis

For the purpose of sensitivity analysis, we also handled the albumin-corrected calcium level as a categorical variable (six subgroups) and found the same trend (p for the trend was 0.003). It is important to note that, compared with the ≥8.5–<9.5 mg/dl group, the risk of 30-day in-hospital mortality will increase by 20% (OR=1.2, 95%CI 1.1–1.3, P<0.001) and 10% (OR=2.1, 95%CI 1.0–4.3, P=0.052) in ≥10.3–< 12 mg/dl and ≥12 mg/dl albumin-corrected calcium subgroups, respectively, which demonstrated the stability of the results (**Table 3**). Furthermore, we used GAM analysis to insert the continuous adjustment variables into the equation by curve fitting. This was generally consistent with model II in the fully adjusted model (OR=1.1, 95% CI 1.0-1.1, P=0.004), which demonstrates the stability of the results (**Table 3**).

Moreover, we conducted sensitivity analyses–excluded patients with cancer, the results showed that albumin-corrected calcium was still positively associated with 30-day in-hospital mortality in the fully adjusted model (OR=1.1, 95% CI 1.0–1.1, P=0.005). We also excluded the ESRD patients for sensitivity analyses. The results indicated that albumin-corrected calcium remained positively associated with 30-day in-hospital mortality with the fully adjusted model (OR=1.1, 95% CI 1.0–1.1, P=0.013) (**Supplementary Table 2**). These sensitivity analysis results suggested that the relationship between albumin-corrected calcium and 30-day in-hospital mortality was very stable.

### The non-linear relationship between albumin-corrected calcium level and 30-day in-hospital mortality

**Figure 4:**
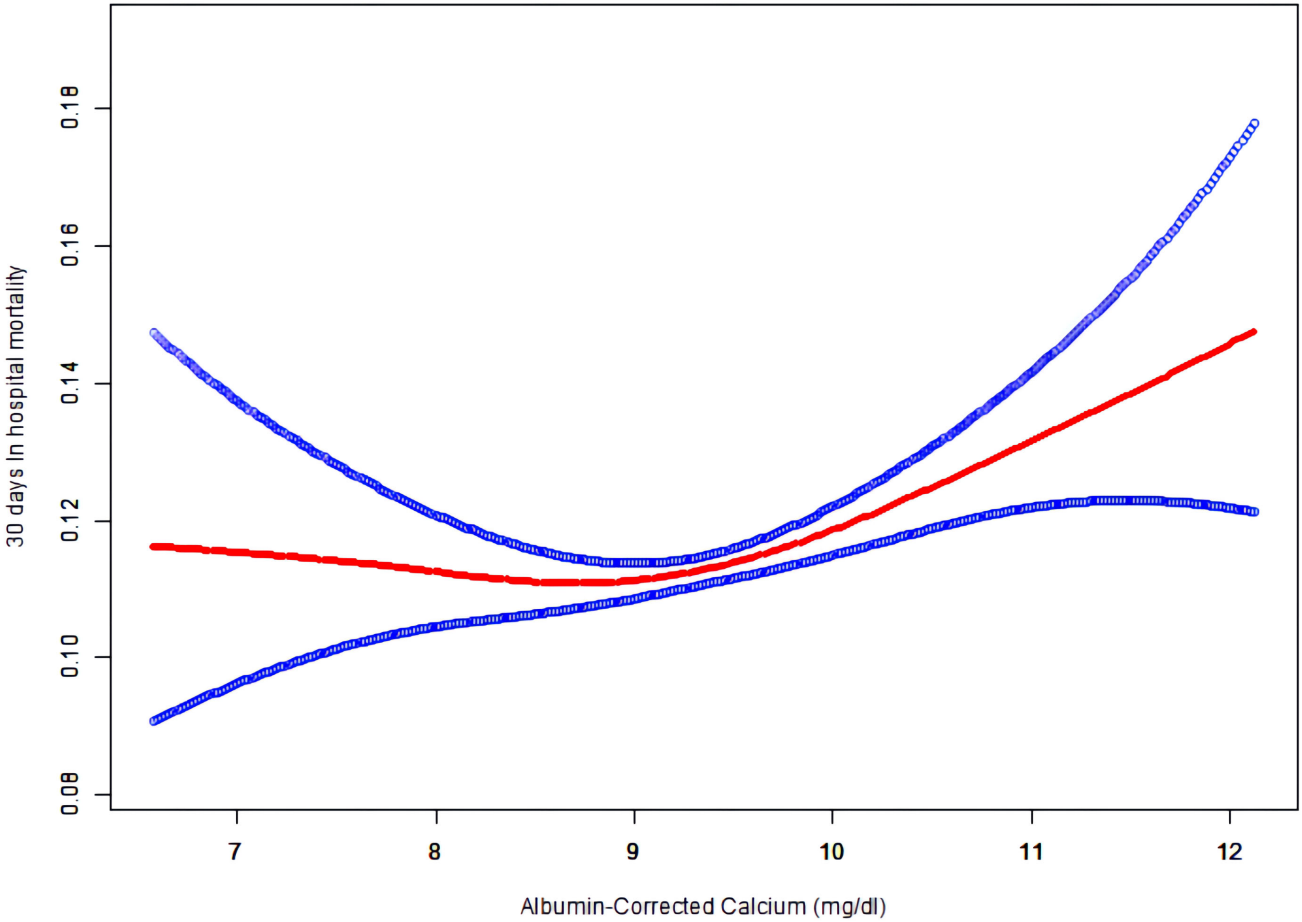
The non-linear relationship between albumin-corrected calcium and 30 days In-hospital mortality.

As albumin-corrected calcium is a continuous variable, we used GAM and smooth curve fitting to explore the relationship between albumin-corrected calcium and 30-day in-hospital mortality. We observed a non-linear dose–response relationship between albumin-corrected calcium and 30-day in- hospital mortality after adjusting these confounding variables (age, gender, BMI, ethnicity, cardiac arrest, gastrointestinal bleeding, diabetes mellitus, cancer, lactic acid, ALT, and serum albumin). The relationship between albumin-corrected calcium and 30-day in-hospital mortality was U shaped (**Figure 4**). Next, we calculated that the inflection point was 8.9 by using a two-piecewise linear regression model (log likelihood ratio test P = 0.005). On the left of the inflection point, the effect size was 0.9 (95% CI 0.8–1.0; P = 0.094), when the albumin-corrected calcium level was <8.9; the risk of 30-day in-hospital mortality decreases by 10% for every 1 mg/dl increase in the albumin-corrected calcium level. On the right of inflection point, the effect size was 1.1 (95% CI 1.1–1.2; P < 0.001), when the albumin-corrected calcium level was ≥8.9; the risk of 30-day in-hospital mortality will increase by 10% for every 1 mg/dl increase in the albumin-corrected calcium level (**Table 1**).

### Relationship between albumin-corrected calcium level and 30-day in-hospital mortality in subgroup analyses

Stratified and subgroup analyses were performed to determine whether age, gender, BMI, ethnicity, cardiac arrest, gastrointestinal bleeding, diabetes mellitus, cancer, lactic acid, ALT, and serum albumin influenced the relationship between the albumin-corrected calcium level and 30-day in- hospital mortality. The results showed that none of these variables affect the relationship between the albumin-corrected calcium level and 30-day in-hospital mortality (**Table 4**), which indicated that the relationship between the albumin-corrected calcium level and 30-day in-hospital mortality was very solid.

**Table 4:** Effect size of albumin-corrected calcium (mg/dl) and 30-day in-hospital mortality in prespecified and exploratory subgroups.

Characteristic	No. of participants	OR (95%CI)	P-value	P for interaction
Age, years				0.9425
<60	37,818	1.2 (1.2, 1.3)	<0.001	
≥60	64,427	1.3 (1.3, 1.4)	<0.001	
Gender				0.3257
Male	54,878	1.4 (1.4, 1.5)	<0.0001	
Female	47,355	1.3 (1.2, 1.3)	<0.0001	
BMI				0.7764
<18.5	6,298	1.4 (1.3, 1.6)	<0.001	
≥18.5, <23.9	23,162	1.4 (1.3, 1.4)	<0.001	
≥23.9	68,598	1.3 (1.3, 1.4)	<0.001	
Ethnicity				0.0540
Black	11,702	1.3 (1.2, 1.4) <0.001	<0.001	
Asian	1,858	1.4 (1.1, 1.8) 0.009	<0.001	
Caucasian	77,587	1.4 (1.3, 1.4) <0.001	<0.001	
Hispanic	4,213	1.4 (1.2, 1.7) <0.001	<0.001	
White	816	1.8 (1.3, 2.5) <0.001	<0.001	
Other	5,161	1.5 (1.3, 1.7) <0.001	<0.001	
Cardiac arrest				0.6310
No	99,301	1.4 (1.4, 1.5)	<0.001	
Yes	2,944	1.2 (1.1, 1.3)	<0.001	
Gastrointestinal bleeding				0.0845
No	95,023	1.3 (1.3, 1.4)	<0.001	
Yes	1,991	1.6 (1.3, 2.0)	<0.001	
Diabetes mellitus				0.7414
No	90,459	1.4 (1.3, 1.4)	<0.001	
Yes	11,786	1.3 (1.2, 1.4)	<0.001	
Cancer				0.8637
No	100,704	1.4 (1.3, 1.4)	<0.001	
Yes	1,541	1.3 (1.0, 1.5)	0.019	
Serum albumin				0.1416
<3.5	59,154	1.2 (1.2, 1.2)	<0.001	
≥3.5	43,080	1.2 (1.1, 1.3)	<0.001	
ALT				0.2158
<40	72,911	1.4 (1.4, 1.5)	<0.001	
≥40	25,026	1.3 (1.2, 1.4)	<0.001	
Lactate				0.6503
<1.7	21,941	1.3 (1.2, 1.4)	<0.001	
≥1.7	25,371	1.2 (1.2, 1.3)	<0.001	

Note1: The above model was adjusted for age, gender, BMI, ethnicity, cardiac arrest, gastrointestinal bleeding, diabetes mellitus, cancer, lactic acid, ALT, and serum albumin.

CI, confidence interval; OR, odds ratio; BMI: body mass index; ALT, alanine aminotransferase.

## Discussion

In this multicenter retrospective cohort of 102,245 participants, we determined the incidence and distribution of abnormalities of albumin-corrected calcium in ICU patients. Hypocalcemia or hypercalcemia occurred in 17.1% of the ICU patients, of which more than 95% were mild calcium abnormalities. We also clarified the association between the albumin-corrected calcium level and 30-day in-hospital mortality. The risk of 30-day in-hospital mortality will increase by 10% in the ≥7.5–<8.5 mg/dl subgroup or 20% in the ≥10.3–<12 mg/dl subgroup when the albumin-corrected calcium level increase by 1mg/dl after adjusting for age, gender, BMI, ethnicity, cardiac arrest, gastrointestinal bleeding, diabetes mellitus, cancer, lactic acid, ALT, and serum albumin. In our study, the relationship between albumin-corrected calcium and 30-day in-hospital mortality was U shaped; the inflection point was 8.9 mg/dl. On the left of the inflection point, the risk of 30-day in- hospital mortality will decrease by 10% for every 1 mg/dl increase in the albumin-corrected calcium level, while on the right of the inflection point, the risk of 30-day in-hospital mortality will increase by 10% for every 1 mg/dl increase in the albumin-corrected calcium level. After a series of sensitivity analyses, stability existed in this relationship.

The high mortality of ICU patients due to numerous risk factors, of which the disorders of electrolytes, including potassium, sodium, and calcium, is one of the most important risk factors (12). Previous studies have explored the association between serum calcium and mortality in different types of diseases (8, 9, 13–20). In Chinese patients with acute pulmonary thromboembolism, hypocalcemia (total serum calcium) is a risk factor of mid- and long-term mortalities (8). A single-center retrospective study in Israel showed that both increased and decreased total serum calcium levels were associated with the increased risk of in-hospital mortality in patients with acute myocardial infarction (9). A US multicenter retrospective cohort study in patients undergoing peritoneal dialysis or hemodialysis found that hypercalcemia (both uncorrected and albumin-corrected calcium) was predicted to increase the risk of mortality by up to 60% (13). However, the relationship of albumin-corrected calcium and 30-days in-hospital mortality in ICU patients is lacking. In this multicenter retrospective cohort study with 102,245 participants, serum calcium abnormalities were an independent risk factor of 30-day in-hospital mortality; albumin-corrected calcium had a significant correlation with 30-day in-hospital mortality after adjustment for potential confounders (OR=1.1, 95% CI 1.0–1.1). Furthermore, the relationship between them was U shaped, especially, compared with the normal serum calcium group (≥8.5, < 9.5mg/dl), the mild hypercalcemia (≥10.3–<12 mg/dl) subgroup was associated with the increased risk of 30-day in-hospital mortality by 1.2-fold.

However, Wang’s study demonstrated that serum calcium had no independent association with hospital mortality. They analyzed the MIMIC-III v1.3 database, which was a single-center ICU database, and found that the relationship between total serum calcium and hospital mortality followed a ‘‘U”-shaped curve, which was similar with ours, but after being adjusted for numerous clinical characteristics, total serum calcium was not associated with hospital mortality (21). The different results among us may be due to 1) different study populations: they used the MIMIC-III v1.3 database, while we used the eICU-CRD v2.0; 2) different types of serum calcium: they selected total serum calcium without corrections, while we chose the albumin-corrected calcium because total calcium is affected by pH and the serum albumin level, and hypoalbuminemia is very common in ICU patients; 3) different confounding factors: they adjusted age, sex, SPO2, SOFA, SIRS, OASIS, SAPSII, SBP, DBP, heart rate, respiratory rate, chronic heart failure, AKI, liver disease, acute pancreatitis, renal replacement therapy, anion gap, creatinine, chloride, glucose, and hemoglobin. However, in our study, according to the recommendation of the STrengthening the Reporting of OBservational studies in Epidemiology (STROBE) statement (22), the covariances, when added to this model, changed the matched odds ratio by at least 10% and were adjusted; thus, we only adjusted these covariances and traditional variables that were reported to possibly affect the relationship between serum calcium and mortality. Aside from GAM and smooth curve fitting, we used a two-piecewise linear regression model and stratified and subgroup analyses and performed a series of sensitivity analyses, and finally, the results between albumin-corrected calcium and 30-day in-hospital mortality were very stable.

There are some advantages in our study. First, this study was a multicenter observational cohort study in a relatively large sample of ICU patients in the USA. Second, a U-shaped relationship between albumin-corrected calcium level and 30-day in- hospital mortality was discovered in our study, and we also calculated the infection point at the same time. Third, to ensure the stability of the results, we performed a series of sensitivity analyses, including converting albumin-corrected calcium to categorical variables, inserting continuous covariates into the equation in the form of a curve by using GAM analysis, and exploring the association between albumin-corrected calcium and 30-day in-hospital mortality after excluding the participants with cancer or ESRD; Fourth, subgroup analysis was perform to ensure the relationship between albumin-corrected calcium and 30-day in- hospital mortality is robust among different participants, and the results are robust. Therefore, our findings may be applied to the treatment of patients with calcium abnormalities.

This study has some limitations. First, our study only focuses on the ICU patients of the USA; thus, the findings in our research may not be applicable to the patients in other departments and of different regions and ethnicities. Second, this study is a retrospective observational study; thus, the association between albumin-corrected calcium and 30-day in-hospital mortality in ICU patients needs to be confirmed in a prospective study. Third, there were no data on the usage of calcium supplements, vitamin D receptor activators, and other medications that may affect calcium metabolism; therefore, the risk of death associated with hypercalcemia may be affected.

## Conclusions

Our data showed that the prevalence of calcium abnormalities in ICU patients was 17.1%, of which more than 95% were mild hypercalcemia and mild hypocalcemia. What is more noteworthy is that even mild hypocalcemia (≥7.5–<8.5 mg/dl) or hypercalcemia (≥10.3–<12 mg/dl) has predicted to increase 10%–20% risk of 30-day mortality when the albumin-corrected calcium level increases by 1 mg/dl in ICU patients. Additionally, the relationship between albumin-corrected calcium and 30-day in-hospital mortality was U shaped; the inflection point was 8.9 mg/dl. Therefore, we clinicians need to pay much more attention to calcium abnormalities and to treat mild hypocalcemia and hypercalcemia timely to improve the patient’s prognosis. In this way, the clinical outcomes of these ICU patients may be significantly improved.

## Data availability statement

The original contributions presented in the study are included in the article/**Supplementary Material**. Further inquiries can be directed to the corresponding authors.

## Ethics statement

Ethical review and approval was not required for the study on human participants in accordance with the local legislation and institutional requirements. Written informed consent for participation was not required for this study in accordance with the national legislation and the institutional requirements.

## Author contributions

RC, QW, and ZW designed the study and wrote the manuscript. RC, XQ, JC, HH, and XC performed the statistical analysis. All authors participated in writing and revising the manuscript and read and approved the final manuscript.
