# Supplementary material for: Non-linear relationship between albumin-corrected calcium and 30-day in-hospital mortality in ICU patients: A multicenter retrospective cohort study
Source: Front Endocrinol (Lausanne). 2022 Dec 21;13:1059201. doi: 10.3389/fendo.2022.1059201 (PMC9810799; doi:10.3389/fendo.2022.1059201)
Supplement: Supplementary file 1 [file DataSheet_1.pdf]

Supplementary Materials

Supplementary Figure 1: Histogram depicting mean albumin-corrected calcium levels layered according to sex and age categories

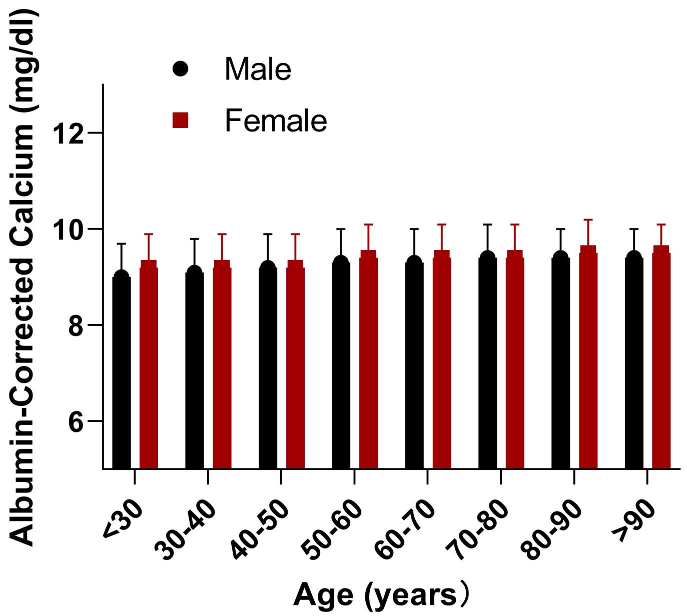

No. of participants

**Male**	2330	2600	4059	7895	9787	10293	8410	2384
**Female**	2324	2999	5320	10609	13214	11742	7540	1565

Supplementary Table 1: Univariate analysis of 30-days In-hospital mortality

30-days In-hospital mortality	Statistics	OR (95%CI)	P-value
Age, years	63.7 ± 16.9	1.0 (1.0, 1.0)	<0.001
Gender			
Male	55313 (53.7%)	Ref.	
Female	47758 (46.3%)	1.0 (0.9, 1.0)	0.241

BMI	27.7 ± 7.7	1.0 (1.0, 1.0)	<0.001
Ethnicity			
Black	11820 (11.6%)	Ref.	
Asian	1874 (1.8%)	1.1 (1.0, 1.4)	0.151
Caucasian	78205 (76.5%)	1.1 (1.0, 1.2)	0.001
Hispanic	4241 (4.2%)	1.2 (1.0, 1.4)	0.009
White	820 (0.8%)	1.0 (0.8, 1.3)	0.993
Other	5213 (5.1%)	1.0 (0.9, 1.1)	0.713
APACHE-IV score	46.6 ± 13.9	1.1 (1.1, 1.1)	<0.001
Diabetes mellitus	11909 (11.6%)	1.1 (1.0, 1.2)	0.007
Ketoacidosis	3470 (3.4%)	0.2 (0.2, 0.3)	<0.001
Hypertension	14015 (13.6%)	0.8 (0.8, 0.9)	<0.001
Acute respiratory failure	16489 (16.0%)	3.6 (3.4, 3.8)	<0.001
COPD	8211 (8.0%)	1.2 (1.1, 1.3)	<0.001
Acute Myocardial Infarction	5761 (5.6%)	1.1 (1.0, 1.3)	0.004
Atrial fibrillation	9303 (9.0%)	1.9 (1.8, 2.1)	<0.001
Cardiac arrest	3007 (2.9%)	9.6 (8.9, 10.4)	<0.001
Congestive heart failure	9564 (9.3%)	1.5 (1.4, 1.6)	<0.001
Gastrointestinal bleeding	2009 (1.9%)	1.0 (0.9, 1.2)	0.603
CKD	1430 (1.4%)	1.6 (1.3, 1.8)	<0.001
ESRD	3288 (3.2%)	1.4 (1.2, 1.6)	<0.001

Sepsis	16289 (15.8%)	2.2 (2.1, 2.3)	<0.001
Stroke	5825 (5.7%)	1.8 (1.7, 2.0)	<0.001
Cancer	1564 (1.5%)	2.0 (1.7, 2.3)	<0.001
Hemoglobin	12.1 ± 2.6	0.9 (0.9, 0.9)	<0.001
Platelet count	226.9 ± 93.6	1.0 (1.0, 1.0)	<0.001
Serum albumin	3.3 ± 0.7	0.4 (0.4, 0.4)	<0.001
Serum creatinine	1.7 ± 2.0	1.1 (1.0, 1.1)	<0.001
Serum magnesium	1.9 ± 0.4	1.3 (1.2, 1.3)	<0.001
Cholesterol	150.1 ± 47.4	1.0 (1.0, 1.0)	<0.001
Triglyceride	139.9 ± 103.1	1.0 (1.0, 1.0)	0.023
ALT	42.2 ± 68.1	1.0 (1.0, 1.0)	<0.001
PH	7.4 ± 0.1	0.2 (0.1, 0.2)	<0.001
Lactate	2.4 ± 1.8	1.2 (1.2, 1.2)	<0.001
Mechanical Ventilation	24772 (24.0%)	4.3 (4.1, 4.5)	<0.001
Nitroglycerin	2892 (2.8%)	0.5 (0.4, 0.6)	<0.001
Glucocorticoids	6393 (6.2%)	1.5 (1.3, 1.6)	<0.001
Vancomycin	5873 (5.7%)	2.2 (2.0, 2.3)	<0.001
Cabapenem	1086 (1.1%)	2.5 (2.1, 2.9)	<0.001
Levofloxacin	2863 (2.8%)	1.7 (1.5, 1.9)	<0.001

Abbreviations: CI, confidence interval; OR, odds ratio; BMI: Body Mass Index; APACHE-IV score: Acute Physiology And Chronic Health Evaluation-IV score;

COPD: Chronic Obstructive Pulmonary Diseases;CKD:Chronic kidney disease; ESRD:End Stage Renal Disease; ALT: alanine aminotransferase.

Supplementary Table 2: Relationship between albumin-corrected calcium(mg/d) and 30-days In-hospital mortality in different sensitivity analyses.

Variable	Model I (OR,95%CI,P)	Model II (OR,95%CI,P)
Alb-Ca (mg/d)	1.1 (1.0, 1.1) 0.005	1.1 (1.0, 1.1) 0.013
Alb-Ca(tertiles)		
<7.5	1.0 (0.7, 1.4) 0.962	0.9 (0.6, 1.3) 0.560
≥7.5, < 8.5	1.2 (1.0, 1.3) 0.023	1.1 (1.0, 1.3) 0.043
≥8.5, < 9.5	Ref	Ref
≥9.5, < 10.3	1.1 (1.0, 1.1) 0.086	1.0 (1.0, 1.1) 0.236
≥10.3,< 12	1.2 (1.1, 1.3) <0.001	1.2 (1.1, 1.3) 0.001
≥12	2.0 (0.9, 4.4) 0.074	2.4 (1.1, 5.0) 0.021
P for trend	0.002	0.011

Model I was sensitivity analysis after excluding those with cancer. we adjust age; gender;BMI;Ethnicity; Cardiac arrest;Gastrointestinal bleeding; Diabetes mellitus; Lactic acid; ALT; Serum albumin.

Model II was sensitivity analysis after excluding ESRD patients. we adjust age; gender;BMI;Ethnicity; Cardiac arrest;Gastrointestinal bleeding; Diabetes mellitus; Cancer; Lactic acid; ALT; Serum albumin.

Abbreviations: OR:odds ratio; CI: confidence interval; Ref: reference; Alb-Ca: Albumin-corrected calcium; ALT: glutamic pyruvic transaminase.
